# The Use of START/STOPP Criteria for Elderly Patients in Primary Care

**DOI:** 10.1155/2013/165873

**Published:** 2013-06-12

**Authors:** Muhteşem Erol Yayla, Uğur Bilge, Elif Binen, Ahmet Keskin

**Affiliations:** ^1^Afyon 5th Primary Care Center, Turkey; ^2^Osmangazi University, School of Health, Meselik, Eskisehir, Turkey; ^3^Adana Sarıçam Türkan Gizer Primary Care Center, Turkey; ^4^Ankara Çankaya 5th Primary Care Center, Turkey

## Abstract

*Aim*. Our aim was to detect older patients who were prescribed inappropriate drugs according to START/STOPP criteria in primary care. *Materials and Method*. Patients aged over 65, admitted to health center no. 5 in Afyon, were included. The files of the subjects were surveyed retrospectively for the final one year in the digital environment, using the Family Medicine Information System. The files surveyed allowed us to list the drugs they used in the past year and to detect inappropriate drug use. *Results*. The number of patients that took part in this study was 325 (average age: 73.23 ± 6.44 years). We found that, among these participants, 48 patients (14.8%) were using drugs inappropriately according to STOPP criteria. *Conclusion*. Further focus on avoiding inappropriate drug use will allow clinicians and other health professionals to reduce side effects and other complications. In patients aged over 65, there is a need to attach particular importance to inappropriate drug use, drug interactions, and avoidance of side effects.

## 1. Introduction

As populations get older (as life expectancy increases in a community), the rate of chronic diseases rises, and hence the amount and diversity of drugs used grow. Although the over 65 population constitutes approximately 13% of the total population, 30% of total medication is prescribed for this age group [1]. Chronic diseases and increasing drug use in the elderly population increase the risk of inappropriate drug use. Inappropriate drug use is an important health issue since it has negative effects on patients and raises health insurance costs [2]. 

 This increasing risk of inappropriate drug use, together with the prevalence of chronic diseases, may cause problems such as drug resistance and side effects [3, 4]. Inappropriate drug use is very common among elderly patients and may cause preventable side effects, hospitalization, death, and waste of resources [5–8]. As the number of elderly people increases in the world population, the quality and safety of drug prescribing are becoming a global health service problem [9]. 

 Dr. Mark Beers set the criteria for inappropriate drug use for the first time in 1991, and these criteria were revised in 1997 and 2003 [10–12]. However, in Beers' criteria, there were certain gaps related to routine clinical content. There has been disagreement on the designation of certain drug types such as amitriptyline and nitrofurantoin as inappropriate drugs, and about half of the drugs prescribed inappropriately are not included in the European Drug Index manuals [13–15]. Furthermore, there is limited research demonstrating tangible benefits of Beers' criteria in terms of clinical outcomes [16]. That is why the applicability and reliability of these criteria are ambiguous. 

The Beers criteria have been used so far to specify potentially inappropriate medications (PIMs) in the elder population. Nevertheless, because the Beers list of criteria poses some serious problems and doubts have been raised about its use in geriatric pharmacotherapy, new criteria have been defined and validated for the identification of inappropriate drugs for elder people. These new criteria are known as STOPP (screening tool of older persons' prescriptions) and START (screening tool to alert to right treatment) [17]. 

 In order to set well-defined criteria and fill in the gap in this field, 18 geriatric pharmacotherapy specialists from Ireland and the United Kingdom accepted START/STOPP criteria on the basis of the Delphi consensus method in 2008. STOPP involves a 65-item list with drug-drug and drug-disease interaction, therapeutic duplication, and increased risk of cognitive deterioration. On the other hand, START is a list of 22 rules related to common instances of prescribing omissions for older people. These criteria apply to patients aged over 65 [18]. 

 The purpose of this study was to detect older patients who were prescribed inappropriate drugs, according to START/STOPP criteria, in their primary care. 

## 2. Materials and Method

The participants in this study were patients aged over 65 who presented to health center no. 5 in Afyon, Turkey. The patients were involved in this study based on simple random sampling and the principle of voluntariness. The patients were first asked about their age and accompanying diseases. The files of participants were surveyed retrospectively for the final one year in the digital environment, using the Family Medicine Information System. The files surveyed allowed us to list the drugs they had used in the past year and to detect inappropriate drug use. START/STOPP criteria were used to identify inappropriate prescriptions [17].

 We first determined the groups of inappropriate drugs and calculated their proportion. The patients were informed about inappropriate drugs they were using and possible side effects. For the purpose of this study, we received approval from the local board of ethics. 

## 3. Results

The number of patients that took part in this study was 325 (average age: 73.23 ± 6.44 years). We found that, among these participants, 48 patients (14.8%) were using drugs inappropriately according to STOPP criteria. There was no indication of drug prescription omissions according to START criteria. Of the patients using drugs inappropriately (average age: 71.06 ± 6.00 years), 22 were male (54.2%) and 26 were female (45.8%); 18 patients (37.5%) were illiterate and 30 patients (62.5%) were literate (only able to read and write) or held a primary school diploma. The average number of diseases in these patients was 2.37 (range was 0–7), and patients with 1 to 3 diseases constituted 83.3% (*n* = 40) of the group. 

The results showed that the most common inappropriately used drug type was NSAID (nonsteroidal anti-inflammatory drugs); 64.6% of the patients used two or more NSAIDs. The second most common inappropriate drug use was related to ASA (acetylsalicylic acid) drugs at a dose of 150 mg/day. The other inappropriate drug prescriptions are provided in Table 1. 

## 4. Discussion

The studies on inappropriate drug use have produced various results. Ay et al. used Beers' criteria in a face-to-face survey with 1019 participants in order to investigate inappropriate drug use and found that the rate of inappropriate drug use was 9.8% [19]. The rate was 12.5% in Finland [20] and was found to be 24.8% in the United Kingdom in a study based on Beer' criteria and conducted in 131 family medicine centers with 162,000 patients [21]. In Taiwan, 882 patients presenting to family medicine or internal diseases policlinics participated in a study which revealed that 97 patients aged over 65 (10.5%) used inappropriate drugs according to Beers' criteria [22]. 

 In our study, the rate of inappropriate drug use was found to be 14% (45 out of 325 patients). As START STOPP criteria are used in our study, it is inevitable that the results would be different from those found in previous research. However, as reported above, the studies that used Beers' criteria have also produced results that were quite different from one other. 

 The present study has shown that two analgesics were used extensively by older patients. With aging, as pain prevalence increases due to osteoarthritis, osteoporosis, and other diseases of the muscular and skeletal system, pain becomes a greater problem for older people [23]. It is expected that in this age group the frequency of various side effects, including renal and gastrointestinal side effects, may increase with the use of NSAIDs. As it has been shown, inappropriate NSAID use may cause gastric irritation, ulcer, chronic blood loss, anemia and sodium retention, and renal failure in patients aged over 65. The effectiveness of antihypertensive drugs may be reduced due to nephrotoxicity [2, 24, 25]. Chronic pain has adverse effects on life quality, physical functions, and wellbeing of old people. Although the use of NSAIDs improves the life quality of these patients, there is a need to consider the risk of renal damage as well as gastrointestinal bleeding and other side effects. That is why clinicians are particularly required to control the renal functions of patients before prescribing drugs [24]. Furthermore, the two analgesics found to be prescribed together may result in an increase in side effects of analgesics due to drug metabolisms changing with aging.

 The combined use of NSAID, ASA, and warfarin may cause particularly increased gastrointestinal system bleeding. It is suggested that the combined use of these drugs is to be avoided; when combined use is essential, they should be consumed with an H_2_ receptor blocker or PPI. In the present study, we found that an H_2_ receptor blocker or PPI was added to the treatment of some patients using warfarin and/or ASA. This is important in order to avoid side effects [17]. 

Dr. Beer's study is of particular importance in that it is the first attempt to compile and organize the drugs that pose risks to older patients. However, due to certain flaws of the list, it cannot be used commonly in Europe. For example, some drugs in the list are not available any more in Europe, or according to more up-to-date data, some drugs are not contraindicated in older people. Among these drugs are amitriptyline, nitrofurantoin, amiodarone, doxazosin, and propranolol. In addition, the criteria defined by Beers do not include information on drug-drug interactions and drug prescription duplication. Furthermore, the Beers criteria do not consider prescribing omission errors that are as important as commission errors in drug appropriateness. The Beers criteria have not been used as a reference for drug appropriateness and minimization of side effects in “prospective randomised controlled trials.” Although the Beers criteria have been largely cited in the literature, it has not been used significantly in clinical studies. The STOPP/START criteria, developed and validated in 2003, are the most recent tool used for the same purpose. The major disadvantage of the STOPP/START criteria is that the references cited are mostly review articles not clinical studies [17]. Besides this, although the START/STOPP criteria provide a useful tool for detecting inappropriate drug use in elderly patients, they cannot replace the clinical judgement of the physician. Moreover, a regular updating of the START/STOPP criteria is essential.

 Further focus on avoiding inappropriate drug use will allow clinicians and other health professionals to reduce side effects and other complications [25]. In patients aged over 65, there is a need to attach particular importance to inappropriate drug use, drug interactions, and avoidance of side effects. The present study may play a guiding role in research in Turkey on the detection of inappropriate drug use with START/STOPP criteria in patients aged over 65. There is still a need for studies using these criteria, which cover longer periods and larger patient populations. 

## Figures and Tables

**Table 1:** Number and percentage of drug inappropriateness according to STOPP criteria.

Drug inappropriateness	*n* (no.)	% (perc.)
Therapeutic (NSAID) duplication	31	64.6
Use of acetylsalicylic acid at dose >150 mg per day	9	18.8
Therapeutic (NSAID) duplication and acetylsalicylic acid at dose >150 mg per day	2	4.2
Use of NSAID in heart failure and therapeutic (NSAID) duplication	1	2.1
Aspirin and warfarin combination without H_2_ receptor blocker or proton pump inhibitor (PPI) and use of acetylsalicylic acid at dose >150 mg per day	1	2.1
Aspirin and warfarin combination without H_2_ receptor blocker or proton pump inhibitor	1	2.1
Use of warfarin in combination with NSAID (increased risk for gastrointestinal bleeding)	1	2.1
Use of calcium channel blocker in chronic constipation	1	2.1
Use of beta_2_ agonist or anticholinergic for mild-mediate chronic obstructive pulmonary disease (COPD) or asthma	1	2.1

Total	48	100.0
